# Self-Microemulsifying Drug Delivery System of Phillygenin: Formulation Development, Characterization and Pharmacokinetic Evaluation

**DOI:** 10.3390/pharmaceutics12020130

**Published:** 2020-02-03

**Authors:** Lingzhi Wang, Wenrui Yan, Yurun Tian, Huanhuan Xue, Jiankai Tang, Liwei Zhang

**Affiliations:** 1 Institute of Molecular Science, Key Laboratory of Chemical Biology and Molecular Engineering of Ministry of Education, Shanxi University, Taiyuan 030006, China; 18834151061@163.com (L.W.); 18404984159@163.com (W.Y.); XHH950302@163.com (H.X.); tjk19834523372@163.com (J.T.); 2 College of Chemistry and Chemical Engineering, Shanxi University, Taiyuan 030006, China; tyr15536502956@163.com

**Keywords:** phillygenin, self-microemulsifying drug delivery system, pharmacokinetics, oral bioavailability, in vitro dissolution

## Abstract

Phillygenin, as an active ingredient of *Forsythia suspensa*, possesses a wide range of biological and pharmacological activity. However, its development and application are restricted due to its poor bioavailability and low solubility. Our work aimed to develop a self-microemulsifying drug delivery system to improve the oral bioavailability of phillygenin. The composition of the self-microemulsifying drug delivery system was preliminary screened by the pseudo-ternary phase diagram. Subsequently, the central composite design method was employed to optimize the prescription of the self-microemulsifying drug delivery system loaded with phillygenin. The prepared self-microemulsifying drug delivery system of phillygenin was characterized in terms of morphology, droplet size distribution, polydispersity index and stability. Then, the in vitro dissolution and the oral bioavailability were analyzed. The optimized self-microemulsifying drug delivery system of phillygenin consisted of 27.8% Labrafil M1944CS, 33.6% Cremophor EL, 38.6% polyethylene glycol 400 (PEG-400) and 10.2 mg/g phillygenin loading. The prepared self-microemulsifying drug delivery system of phillygenin exhibited spherical and uniform droplets with small size (40.11 ± 0.74 nm) and satisfactory stability. The in vitro dissolution experiment indicated that the cumulative dissolution rate of the self-microemulsifying drug delivery system of phillygenin was significantly better than that of free phillygenin. Furthermore, after oral administration in rats, the bioavailability of phillygenin was significantly enhanced by the self-microemulsifying drug delivery system. The relative bioavailability of the self-microemulsifying drug delivery system of phillygenin was 588.7% compared to the phillygenin suspension. These findings suggest that the self-microemulsifying drug delivery system of phillygenin can be a promising oral drug delivery system to improve the absorption of phillygenin.

## 1. Introduction

The fruit of *Forsythia suspensa* (Thunb.) Vahl (Oleaceae) is a well-known traditional Chinese medicine (TCM), named “Lianqiao” in Chinese. It is distributed in China, Korea, Japan and many European countries [1]. *Forsythia suspensa* (*F. suspensa*) exhibits anti-bacterial, anti-inflammatory, antioxidant, anti-allergy, anti-virus and anti-cancer effects [2]. So far, more than 230 compounds have been separated and identified from *F. suspensa* [2], including phenylethanoid glycosides [3], flavonoids [4], triterpenoids [5], lignans [6] and other compounds.

Phillygenin (PG), a bisepoxylignan compound [7], is one of the major ingredients in *F. suspensa* and has been used as a marker for the quality control of *F. suspensa* in several studies. Recently, PG has provoked great interest due to its significant pharmacological activities, such as antioxidant, hypolipidemic [8], inhibition of tyrosinase activity [9] and anti-hypertensive effects [10]. Particularly, its remarkable ability to treat liver injury [11] and its anti-inflammatory properties have drawn great attention. Tang [12] found that the anti-inflammatory mechanism of PG was related to the inhibition of the expression of inducible nitric oxide synthase (iNOS) and cyclooxygenase-2 (COX-2). However, the hepatoprotection and anti-inflammatory activity of PG was discounted due to its poor water solubility and rapid metabolism [11]. Until now, few studies have focused on improving the oral bioavailability of PG. Consequently, it is essential to develop a suitable delivery system to successfully overcome these challenges and improve the oral absorption of PG.

Oral formulations are preferred in drug delivery systems because of high patient convenience, good compliance and flexibility in regimen. Nevertheless, some drugs have good pharmacological activities, but their poor aqueous solubility limits their absorption. At present, the methods of enhancing drug solubility mainly include liposomal encapsulation, solid dispersion, macromolecule micelles, cyclodextrin inclusion complexes, nanoemulsion, and self-microemulsifying drug delivery system (SMEDDS) [13]. However, much more attention has been focused on SMEDDS, because SMEDDS, as a carrier system, exhibit splendid biocompatibility, biodegradability, stability, and enhanced permeability [14].

SMEDDS is an isotropic mixture consisting of an oil, surfactant, co-surfactant and the drug [15], which can form fine oil-in-water microemulsion with a droplet size less than 100 nm in aqueous phases by gentle agitation [16,17]. The microemulsion droplets dispersed in the gastrointestinal tract provide a large surface area and promote rapid release of the drug, which is beneficial to improve the absorption and bioavailability of the drug. Overall, SMEDDS, as an efficient drug delivery system, not only protects unstable drugs but allows these drugs to quickly exert their effects [18]. SMEDDS can be used as an excellent carrier for hydrophobic, poor absorbing and easily hydrolyzable drugs [19,20]. In recent years, SMEDDS has been extensively investigated for improving the oral bioavailability of unstable and water insoluble drugs, such as Ligusticum chuanxiong oil [21], curcumin [22], resveratrol [23] and simvastatin [24]. To the best of our knowledge, the formulation of phillygenin-loaded self-microemulsifying drug delivery system (PG-SMEDDS) has not been developed. Therefore, the objective of the current research was to optimize and prepare PG-SMEDDS. In addition, the physicochemical characterizations of PG-SMEDDS were conducted. Furthermore, in vitro dissolution of PG-SMEDDS in different dissolution media and the in vivo oral bioavailability were both evaluated.

## 2. Materials and Methods

### 2.1. Materials and Chemicals

PG reference substance (>98%) and kaempferol (internal standard (IS)) were both obtained from Chengdu Must Bio-Technology Co., Ltd. (Chengdu, China). PG (laboratory self-prepared, purity: 98.9%), olive oil, Tween 80 (TW80) and isopropyl myristate (IPM) were all purchased from Shanghai Macklin Biochemical Co., Ltd. (Shanghai, China). P-octyl polyethylene glycol phenyl ether (OP-10 emulsifier) was purchased from Aladdin Industrial Co., Ltd. (Shanghai, China). Cremophor RH40 and Cremophor EL (EL-35) were both supplied by BASF Corporation (Ludwigshafen, Germany). Transcutol HP, medium chain triglycerides (MCT), and Labrafil M1944CS were received as gifts from Gattefosse (Saint-Priest Cedex, France). Ethyl oleate, 1,2-propanediol, polyethylene glycol 400 (PEG-400) and glycerol were gifted from Sinopharm Chemical Reagent Co., Ltd. (Shanghai, China). Acetonitrile and methanol (High Performance Liquid Chromatography (HPLC) grade) were purchased from Fisher Co., Ltd. (Waltham, MA, USA). All other solvents or reagents used were of analytical grade. Distilled water was used for all relevant experiments.

### 2.2. High Performance Liquid Chromatography (HPLC) Analysis of PG

#### 2.2.1. HPLC Conditions

PG samples were analyzed using an Agilent 1260 HPLC system (Agilent, Santa Clara, CA, USA). Chromatographic separation and analysis were carried out using a Venusil XBP C18 column (250 × 4.6 mm, 5 μm particle size, Agela Technologies., Tianjin, China) at a column temperature of 30 °C. The UV detection wavelength was set at 277 nm. The mobile phase consisted of methanol and 0.3% glacial acetic acid aqueous solution at the ratio of 65:35 (*v*/*v*). The flow rate was 1.0 mL/min and the injection volume was 10 μL.

#### 2.2.2. Preparation of the Standard and Sample Solutions

The PG reference substance was dissolved in methanol to prepare the stock solution of 2.03 mg/mL and stored at 4 °C. The test solution was prepared by dissolving 0.5 g of PG-SMEDDS in 10 mL of methanol. Similarly, the blank solution was prepared using SMEDDS without PG according to the preparation method of the test solution. All samples were filtrated through a 0.45 μm membrane filter before HPLC analysis.

#### 2.2.3. Method Validation

HPLC method validation was carried out by determining linearity, specificity, precision, repeatability, stability and accuracy. The selectivity was studied by comparing the test sample solution, blank solution and the reference solution to evaluate the interference of the excipients. To establish the linearity and range, a series of concentration levels (0.05, 0.1, 0.2, 0.4, 0.6, 0.8 and 1.0 mg/mL) were determined for solubility studies. Linearity was described by the regression line equation and its correlation coefficient. Precision was validated by injecting six repeats of the sample solution. To confirm repeatability, six different test solutions from the same sample were injected. To evaluate stability, the same working solution was analyzed after preparation for 0, 2, 4, 6, 8, 12 and 24 h. The recovery was determined by spiking samples with a known amount of PG reference substance. Another series of samples with low concentration levels (0.005, 0.01, 0.02, 0.04, 0.06, 0.08 mg/mL) were also determined for the dissolution study, and linearity together with range were established.

### 2.3. Solubility Studies

In order to select proper components for the preparation of SMEDDS, the solubility of PG in various oils, surfactants, and co-surfactants was determined. Briefly, an excess amount of PG was added individually to 1 g of various vehicles and the mixtures were whirled using vortex shaker (GL-88B Vortex mixer, Kylin-Bell Lab Instruments Co., Ltd., Haimen, Jiangsu, China) for 10 min. Then, the mixtures were shaken reciprocally at 37 °C for 48 h in a water-bath shaker (HZS-H, Haerbin Donglian Electronic Technology Development Corporation, Haerbin, China) to reach equilibrium. After that, the mixtures were centrifuged at 10,000 rpm for 15min, and the supernatant was diluted with methanol and filtered through a 0.45 μm membrane filter (Jinteng, Tianjin, China). The concentration of PG in the supernatant was determined using the validated HPLC method.

### 2.4. Self-Emulsifying Grading Test

According to the results from Section 2.3, the chosen oils were added to the chosen surfactants in tubes at different ratios (1:9, 2:8, 3:7, 4:6, 5:5, 6:4, 7:3, *w/w*), respectively, and the mixtures were mixed for 5 min. Then, the mixtures were diluted with distilled water (1:100 *v*/*v*) and magnetically stirred at 37 °C. The emulsification process and final appearance was observed, which had been divided into the following five grades through a visual grading system [25]:

Grade A: time of self-emulsification was less than 1 min, and the emulsion was clear or slightly light blue in appearance. Grade B: time of self-emulsification was less than 2 min, slightly less clear emulsion which has a bluish-white appearance. Grade C: the self-emulsification time was in the range of 1–3 min, the appearance of the emulsion was a bright white opaque liquid. Grade D: the self-emulsification time was more than 3 min and the color of the emulsion was dull. The appearance of emulsion was grayish white and slightly oily. Grade E: it was difficult to emulsify and there were always oil droplets in the emulsion.

### 2.5. Construction of Pseudo-Ternary Phase Diagrams

Pseudo-ternary phase diagrams are a tool for screening suitable components and identifying the well-suited ratios of constituents in SMEDDS [26]. Based on the results of the self-emulsifying grading test, surfactants and oil phase have been determined. At first, the weight ratio of surfactant to co-surfactant (Km) was fixed at 2:1. Subsequently, the mixture (surfactant and co-surfactant) was mixed with the oil phase at different weight ratios from 1:9 to 9:1. One gram of the mixture was titrated with distilled water and stirred in a constant temperature water-bath at 37 °C until the mixture began to form a clear or light blue opalescent liquid, and then the mass fraction of the each of the ingredients was recorded, respectively. Then, Origin 8.0 software (OriginLab Corp., Northampton, MA, USA) [27] was used to construct the pseudo-ternary phase diagrams. The optimum co-surfactant was screened by comparing the region of the microemulsion in pseudo-ternary phase diagram [28].

According to the optimized results of oils, surfactants and co-surfactants, the range of the amount of each ingredient was further studied by means of a pseudo-ternary phase diagram. In order to screen the optimal range of Km, the pseudo-ternary phase diagrams with different Km were plotted. Afterwards, a series of selected Km and oil were mixed homogeneously, and the ratios of oil to Km were varied from 9:1 to 1:9 (*w/w*), respectively. Then, the distilled water was added dropwise to the mixture until the mixture became clear. Finally, pseudo-ternary phase diagrams were constructed to determine the application range of oil phase.

### 2.6. Formulation Optimization of SMEDDS

The pseudo-ternary phase diagram merely determined the range of the prescription, and obtaining the prescription still required further optimization. A two-factor and five-level (±α, 0, ±1) central-composite design (CCD) was adopted to optimize the formulation of PG-SMEDDS [29]. Based on the preliminary experiments, two factors of oil percentage (X_1_) and Km (X_2_) were set as independent variables, and the equilibrium solubility (Y_1_), droplet size (Y_2_) and polydispersity index (PDI) (Y_3_) were selected as the responses because they were generally considered as significant indicators for assessing the qualities of SMEDDS. The factors and levels of experimental design are shown in Table 1.

Different models were selected to fit the responses of Y_1_, Y_2_ and Y_3_ to the variables of X_1_ and X_2_ using Design Expert 8.0.6 software (State-Ease Inc., Minneapolis, MN, USA). The model that had the largest correlation coefficient (r) of the regression was adopted to draw the contour lines and response surfaces. Besides, the effect of independent variables on the dependent variables was predicted by employing response surface methodology.

### 2.7. Characterization of Phillygenin-Loaded Self-Microemulsifying Drug Delivery System (PG-SMEDDS)

#### 2.7.1. Type Identification

Two batches of PG-SMEDDS samples were prepared in parallel and placed in two vials. After PG-SMEDDS was diluted with distilled water, an equal amount of oil-soluble dye (Sudan Red) and water-soluble dye (Methylene Blue) were added. The type of microemulsion was judged by observing the diffusion speed of the two dyes.

#### 2.7.2. Morphological Observation and Droplet Size

The morphology of PG-SMEDDS was observed by transmission electron microscopy (TEM) (JEM-2010, JEOL, Tokyo, Japan). PG-SMEDDS was diluted with distilled water at a ratio of 1:100 (*v*/*v*) at 37 °C, and a drop of diluted sample was deposited on a film-coated copper grid. After standing for ten minutes, the excess liquid was removed by a piece of filter paper. Subsequently, the grid was stained with one drop of 2% aqueous solution of phosphotungstic acid for 5 min and the excess solution was drawn off with filter paper. After natural drying, the morphology of the microemulsion was observed and photographed under TEM at 25 °C. The PG-SMEDDS was diluted (50-fold) with distilled water and gently stirred to mix thoroughly. The droplet size and polydispersity index (PDI) of the SMEDDS were determined using a Zetasizer Nano ZS 90 (Malvern Instruments Ltd., Malvern, Worcestershire, UK) at 25 °C with a scattering angle of 90°.

#### 2.7.3. Stability of PG-SMEDDS

To evaluate the stability of the prepared PG-SMEDDS, the sample was diluted with distilled water, stirred uniformly and sealed in bottles. Then, the samples were stored for one month at 25 °C, 37 °C and 60 °C, respectively [14]. Samples were taken at 10, 20 and 30 days, respectively, and observed for phase separation or drug precipitation.

### 2.8. In Vitro Dissolution Study

A dissolution test was conducted according to the Chinese Pharmacopoeia (Pharmacopoeia Commission of PRC, 2015) Appendix Method I (the basket method) [30]. In order to compare the dissolution behavior of PG-SMEDDS and free PG, 900 mL of distilled water, pH 1.2 HCl, and pH 6.8 phosphate buffer were used as dissolution media. SMEDDS containing 10 mg of PG or 10 mg of free PG were encapsulated in hard gelatin capsules and introduced into the dissolution medium. The dissolution behavior of PG-SMEDDS and free PG was assessed using an automatic dissolution apparatus (DIS-8000, Copley Instruments Ltd., UK) at 100 rpm and 37 ± 0.5 °C. At predetermined time intervals of 3, 5, 10, 15, 20, 30, 45, 60, 90, 120 and 240 min, 5 mL of the dissolution media was removed, while the isothermal and equal volume dissolution medium was supplemented. The sample was filtered through a 0.45 μm membrane filter (Jinteng, Tianjin, China) and the amount of PG was quantified using HPLC. The cumulative dissolution rate of PG was calculated and the dissolution profiles were plotted.

### 2.9. In Vivo Pharmacokinetic Study

#### 2.9.1. Collection of Plasma Samples

Twelve male SD rats (260–280 g) were supplied by the Animal Center, Beijing SPF Biotechnology Co., Ltd. (Beijing, Certificate No. SCXK 2016–0002). The rats were fed under normal laboratory conditions (temperature of 23 ± 1 °C, relative humidity of 50% ± 10%) under a 12 h light-dark cycle. Before the experiment, all rats were fed adaptively for 7 days in the animal room, and a standard pelleted feed and water ad libitum were provided. Then, the rats were randomly and equally divided into two groups. All animals were fasted overnight before starting pharmacokinetic studies with free access to water. Each group of rats was orally administered either PG-SMEDDS or a PG suspension (PG dispersed in 0.5% sodium carboxymethyl cellulose) at a dose of 100 mg/kg. After oral administration, blood samples (approximately 0.5 mL) were collected from the retro-orbital plexus and contained in heparinized microfuge tubes at 0, 0.083, 0.25, 0.5, 1, 2, 3, 4, 6, 8, 10, 12 and 24 h. Subsequently, the blood samples were centrifuged at 3500 rpm for 15 min and the plasma samples were stored at −80 °C until analysis.

All experimental rats were treated according to the guidelines of the Animal Research Committee of Shanxi Institute of Medicine and Life Science, and the experimental procedures were approved by the Animal Ethics Committee of the institution (SYXK 2017-0001). All animal experiments were in compliance with the National Act of the People’s Republic of China on the care and use of laboratory animals.

#### 2.9.2. Ultra Performance Liquid Chromatography (UPLC) Analysis

Kaempferol (50 µL) as an internal standard (20 μg/mL) was added to 200 μL of plasma sample and vortexed for 1 min. All plasma samples were extracted with acetonitrile (800 μL) to precipitate protein and centrifuged at 12,000 rpm for 10 min at 4 °C. The supernatant was dried at 40 °C under a stream of nitrogen. The dried residue was reconstituted with 50 μL of 60% methanol and centrifuged at 13,000 rpm for 15 min. Then, 10 μL of the supernatant was detected by UPLC (Waters, Milford, MA, USA). Acquisition and analysis of data were performed by Empower 2 software (Waters, Milford, MA, USA).

The method described by Song et al. [11] was applied to sample analysis with minor modifications. The chromatographic column used was an ACQUITY UPLC^®^ BEH C_18_ (100 × 2.1 mm, 1.7 μm, Waters, Milford, MA, USA). The column temperature was 40 °C and injection volume was 10 μL. The sample was eluted at a flow rate of 0.3 mL/min and the mobile phase consisted of acetonitrile (A) and 0.1% formic acid water (B). The elution gradient was 10% A at 0–2 min, 10–90% A at 8–12 min, 90–10% A at 12–15 min, and the samples were determined under UV detection at 280 nm.

The linear regression equation of the calibration curve of PG was C = 0.138 A + 0.027 with a correlation coefficient of 0.9990 and the linear range was 0.3–19.2 μg/mL. The specificity and stability of the developed method was satisfactory. When the QC sample concentration was 0.6, 2.4 or 9.6 μg/mL, the relative standard deviations of intra-day and inter-day precision were in the range of 4.40–8.38% and 3.14–5.91%, respectively. The extraction recovery of PG was within the range of 81.03–84.89%. The limit of detection (signal/noise = 3) of PG in rat plasma was 0.08 μg/mL and the lower limit of quantification (signal/noise = 10) was 0.3 μg/mL.

#### 2.9.3. Analysis of Pharmacokinetic Parameters

Drug and Statistics 3.2.8 software (DAS 3.2.8, developed by the China Quantitative Pharmacology Professional Committee) was used for processing data. The non-compartmental model was used for calculating the main pharmacokinetic parameters. The area under the concentration-time curve (AUC) from zero to the last time point was calculated by the linear trapezoidal method. The relative bioavailability (*F*) of PG-SMEDDS to the PG suspension was calculated according to the following equation:

*F* = (AUC_test group_/AUC_reference group_) × 100 (1)

### 2.10. Statistical Analysis

Data were expressed as mean ± standard deviation and analyzed by SPSS version 16.0 software (SPSS Inc., Chicago, IL, USA). Statistical data analyses were performed using one-way analysis of variance (ANOVA). The values of *P* < 0.05 were considered to be statistically significant.

## 3. Results and Discussion

### 3.1. Method Validation Results

The results showed that the method had good specificity (Supplementary Materials Figure S1). At the same retention time (about 6.8 min), there was a corresponding chromatogram between the test solution and the reference solution. There were no interference peaks in the chromatogram of the blank solution, which indicated that the excipients had no interference on the determination of PG. In the concentration range of 0.05–1.00 mg/mL, the linear regression equation was Y = 9225.3X − 82.709 with good linear regression (R^2^ = 0.9996). The relative standard deviations (RSD) of precision and repeatability confirmed the method’s preciseness with 0.12% and 0.93%. The stability test illustrated that samples were stable within 24 h (RSD = 0.36%). Recovery values of 98.82–100.15% demonstrated that the method was accurate. Within the low concentration range of 0.005–0.08mg/mL, the linear regression equation was Y = 8177.4X, which showed good linear regression (R^2^ = 0.9991). Therefore, the HPLC method was reliable to conduct the analysis of PG.

### 3.2. Solubility of PG

The solubility of PG in different vehicles was assayed and the results are presented in Table 2. According to solubility of PG, Labrafil M1944CS and MCT were selected as oil phases, EL-35, OP-10 and Tween 80 were selected as surfactants, and Transcutol HP and PEG-400 were selected as co-surfactants for further screening.

### 3.3. Self-Emulsifying Grading

The visual grading results of the formed microemulsion are shown in Table 3. The mixtures of Labrafil M1944CS and EL-35 mixed at different ratios formed a better microemulsion than other combinations. Thus, Labrafil M1944CS was chosen as the oil phase and EL-35 was chosen as the surfactant.

### 3.4. Pseudo-Ternary Phase Diagrams

**Figure 1:**
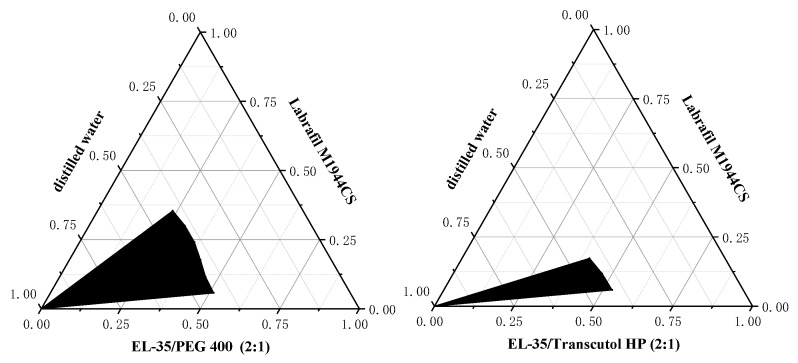
Pseudo-ternary phase diagram of different co-surfactants. The black area represents the microemulsion region, and the white area represents the coarse emulsion region.

Pseudo-ternary phase diagrams provided information on the phase behavior of various compositions in SMEDDS. Therefore, the pseudo-ternary phase diagrams were important for evaluating the self-microemulsifying ability of SMEDDS formulations and determining the range of prescription composition. The pseudo-ternary phase diagrams of PEG-400 and Transcutol HP are shown in Figure 1. Under the same conditions, PEG-400 had the largest microemulsion region compared with Transcutol HP. Consequently, PEG-400 was identified as the most desirable co-surfactant.

**Figure 2:**
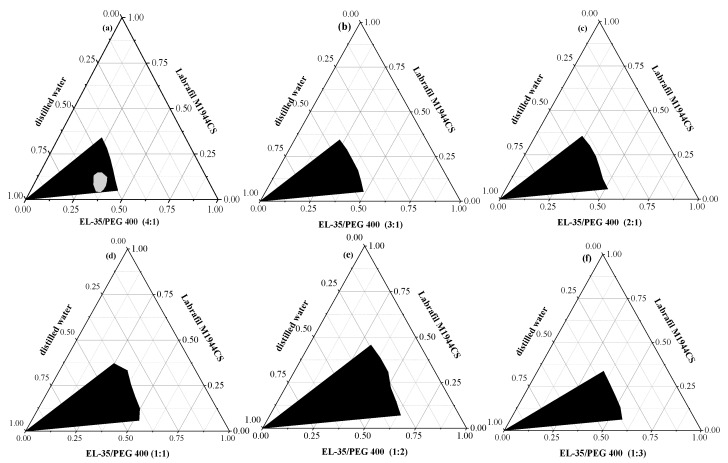
Pseudo-ternary phase diagram of different Km. EL-35:PEG-400 (4:1) (**a**), EL-35:PEG-400 (3:1) (**b**), EL-35:PEG-400 (2:1) (**c**), EL-35:PEG-400 (1:1) (**d**), EL-35:PEG-400 (1:2) (**e**), and EL-35:PEG-400 (1:3) (**f**). The black area represents the microemulsion region, the white area represents the coarse emulsion region, and the grey area represents the gel-like region.

**Figure 3:**
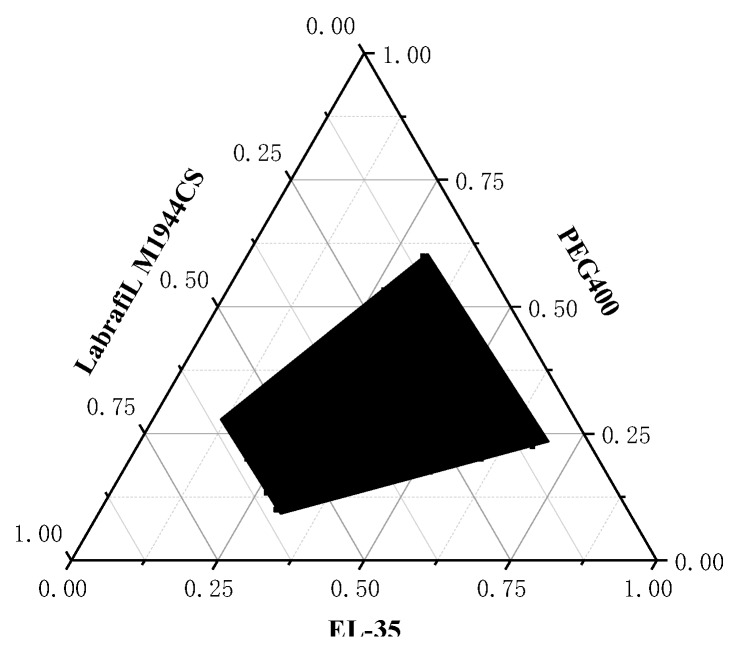
Ternary phase diagram of the blank self-microemulsifying drug delivery system (SMEDDS). The black area represents the microemulsion region, and the white area represents the coarse emulsion region.

The pseudo-ternary phase diagrams of different Km are displayed in Figure 2. When Km > 3, a conspicuous gel zone appeared during the stirring process and the self-emulsification time was prolonged, which would affect the dispersion of the drug. Nevertheless, the microemulsion region began to reduce when Km < 0.5. Taking into account the emulsification efficiency and the clarity of the microemulsion, the range of Km was limited to 0.5–3. Figure 3 shows that the microemulsion cannot be formed when the oil content was higher than 60%. Therefore, the range of the oil phase was limited to 10–60%.

### 3.5. Formulation Optimization of PG-SMEDDS

A two-factor and five-level central-composite design was adopted to optimize the formulation of PG-SMEDDS. The experimental design and results of the equilibrium solubility, droplet size and PDI are listed in Table 4.

The polynomial fitting correlation coefficients (r) of Y_1_, Y_2_ and Y_3_ for the quadratic polynomial were 0.9532, 0.9954 and 0.9777, respectively. Statistical data showed that the *P*-value of the quadratic polynomial model was less than 0.05 (significantly different), and all R^2^ and adjusted R^2^ values of the responses were similar (difference between R^2^ and adjusted R^2^ < 0.2). We could conclude that the relationship between Y (equilibrium solubility, droplet size and PDI) and X (weight percent of oil and Km) fitted well with the quadratic polynomial model. Moreover, all the three responses were fitted to the quadratic polynomial model. The quadratic polynomial equations were represented as follows:

Y_equilibrium solubility_ = 23.77 − 5.26X_1_ − 1.17X_2_ + 4.96X_1_X_2_ − 2.18X_1_^2^ − 4.42X_2_^2^ (r = 0.9532, *P* = 0.0016);

Y_droplet size_ = 51.57 + 23.91X_1_ − 4.48X_2_ − 2.50X_1_X_2_ + 1.25X_1_^2^ − 0.81X_2_^2^ (r= 0.9954, *P* < 0.0001);

Y_PDI_ = 0.29 + 0.012X_1_ + 0.033X_2_ − 0.012X_1_X_2_ − 0.029X_1_^2^ − 0.023X_2_^2^ (r = 0.9777, *P* = 0.0001).

**Figure 4:**
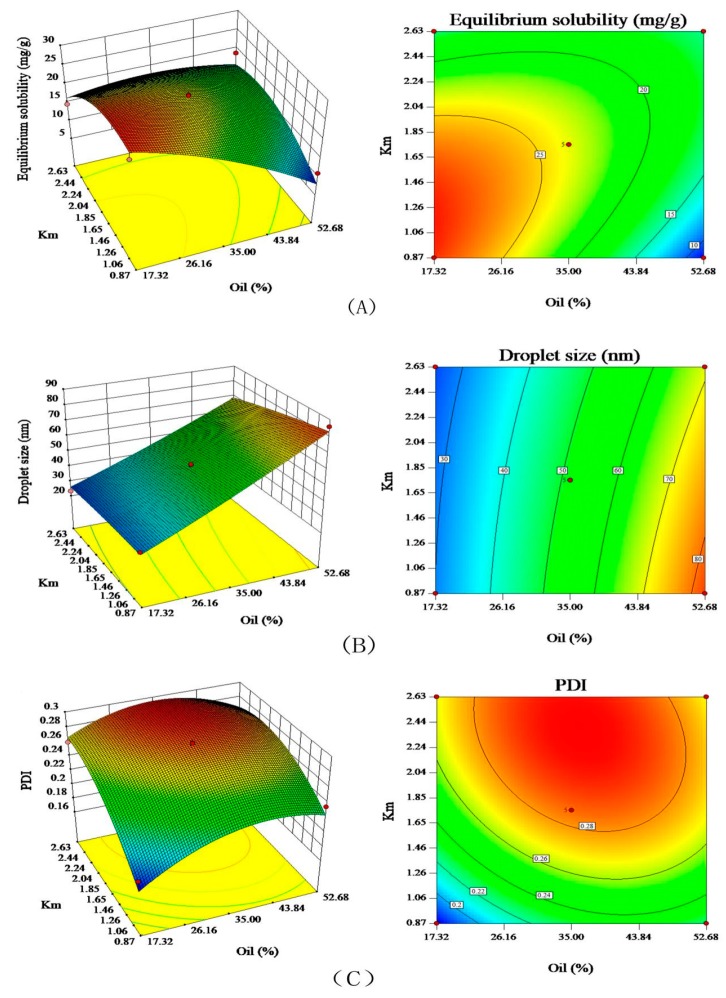
Response surfaces and contour plots of equilibrium solubility, droplet size and PDI. (**A**) equilibrium solubility, (**B**) droplet size, and (**C**) PDI.

The response surface and contour plots visually reflected the interactions between various factors and responses. As shown in Figure 4, increasing the oil percentage led to an increase in droplet size; PDI increased first and then decreased when the Km maintained constant. When Km increased from 0.87 to 1.35, the increase of oil percentage caused a decrease in equilibrium solubility, but the equilibrium solubility first increased and then decreased with Km > 1.35. When oil percentage was constant, the droplet size decreased as Km increased. However, equilibrium solubility and PDI first increased and then decreased with the increase of Km.

Prescription optimization was performed by the predictive optimization function of Design Expert 8.0.6 software with minimum droplet size, minimum PDI and maximum equilibrium solubility as the limiting conditions. The best formulation with a Km of 0.87 and oil percentage of 27.79% was obtained. In other words, the optimized PG-SMEDDS consisted of 27.8% Labrafil M1944CS, 33.6% Cremophor EL and 38.6% PEG-400. The predicted equilibrium solubility was 24.33 mg/g with a droplet size of 40 nm and PDI of 0.241.

#### Validation of Optimization Model

To confirm the adequacy and reliability of the predictive model, three batches of PG-SMEDDS samples were prepared according to the optimal prescription, and the three responses (equilibrium solubility, particle size and PDI) were further evaluated. The results indicated that the experimental equilibrium solubility of PG in SMEDDS was 24.16 ± 0.08 mg/g, and the deviation from the predicted value was 0.70%. The particle diameter was 40.04 ± 0.70 nm and its deviation was 0.10%. The experimental value of PDI was 0.243 ± 0.01 with a deviation of 0.83%. Based on the fine agreement between the predicted and experimental results, the established mathematical model was proven to be reliable.

Further research found that the microemulsion formed by SMEDDS containing 24.16 mg/g of PG had poor stability and phase separation occurred within 20 min. Hence, in order to prevent the PG from precipitating in the gastrointestinal tract, the amount of PG in SMEDDS was further investigated. The results showed that PG precipitation was not observed in the microemulsion with 10.2 mg/g of PG for a long time at room temperature. The encapsulation efficiency of SMEDDS to PG could reach 92%. Based on the above considerations, the amount of PG in the prescription of PG-SMEDDS was determined to be 10.2 mg/g.

### 3.6. Characterization of PG-SMEDDS

#### 3.6.1. Type of Microemulsion

The microemulsion type identification results are presented in Figure S2 (Supplementary Materials). As shown, the water-soluble dye diffused rapidly in the microemulsion, but the oil-soluble dye only deposited without diffusion. The results showed that the type of microemulsion was oil-in-water (o/w).

#### 3.6.2. Characterization of PG-SMEDDS

**Figure 5:**
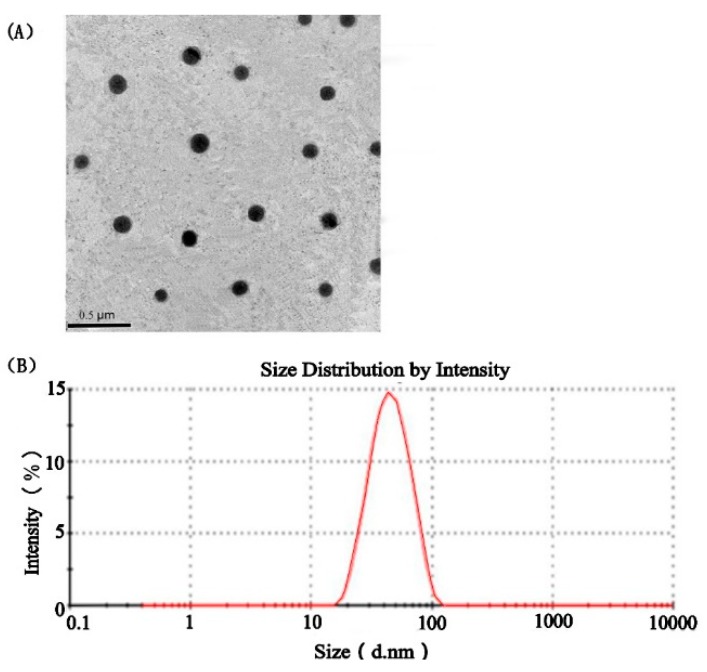
Characterization of the prepared PG formulations. (**A**) Transmission electron microscopy (TEM) image of the optimized phillygenin-loaded self-microemulsifying drug delivery system (PG-SMEDDS); (**B**) droplet size distribution of PG-SMEDDS.

The TEM image (Figure 5A) showed that the microemulsion droplets were spherical and had a uniform shape without aggregation. The droplet size distribution exhibited a narrow range from 15 to 100 nm with an average droplet size of 40.11 ± 0.74 nm (Figure 5B). The droplet size of the microemulsion was one of the most important parameters that affected the release rate and stability of the drug. A smaller droplet size could provide a great interfacial area, which was advantageous for drug absorption in the gastrointestinal tract [31]. The PDI of the microemulsion was 0.151 ± 0.009, which revealed that PG-SMEDDS exhibited wonderful dispersion properties. The small droplet size and good dispersibility of the microemulsion indicated that the bioavailability and absorption of PG can be improved by SMEDDS.

#### 3.6.3. Stability of PG-SMEDDS

The optimized PG-SMEDDS exhibited desirable stability during the test period. After storage at 25 °C and 37 °C for one month, the PG-SMEDDS was still clear and transparent without any phase separation or drug precipitation. After standing at 60 °C for 10 days, a slightly turbidity was observed in the microemulsion, but it could return to clear and transparent after being left at room temperature for a while. Hence, the results indicated that SMEDDS exhibited satisfactory stability.

#### 3.6.4. In Vitro Dissolution Study

**Figure 6:**
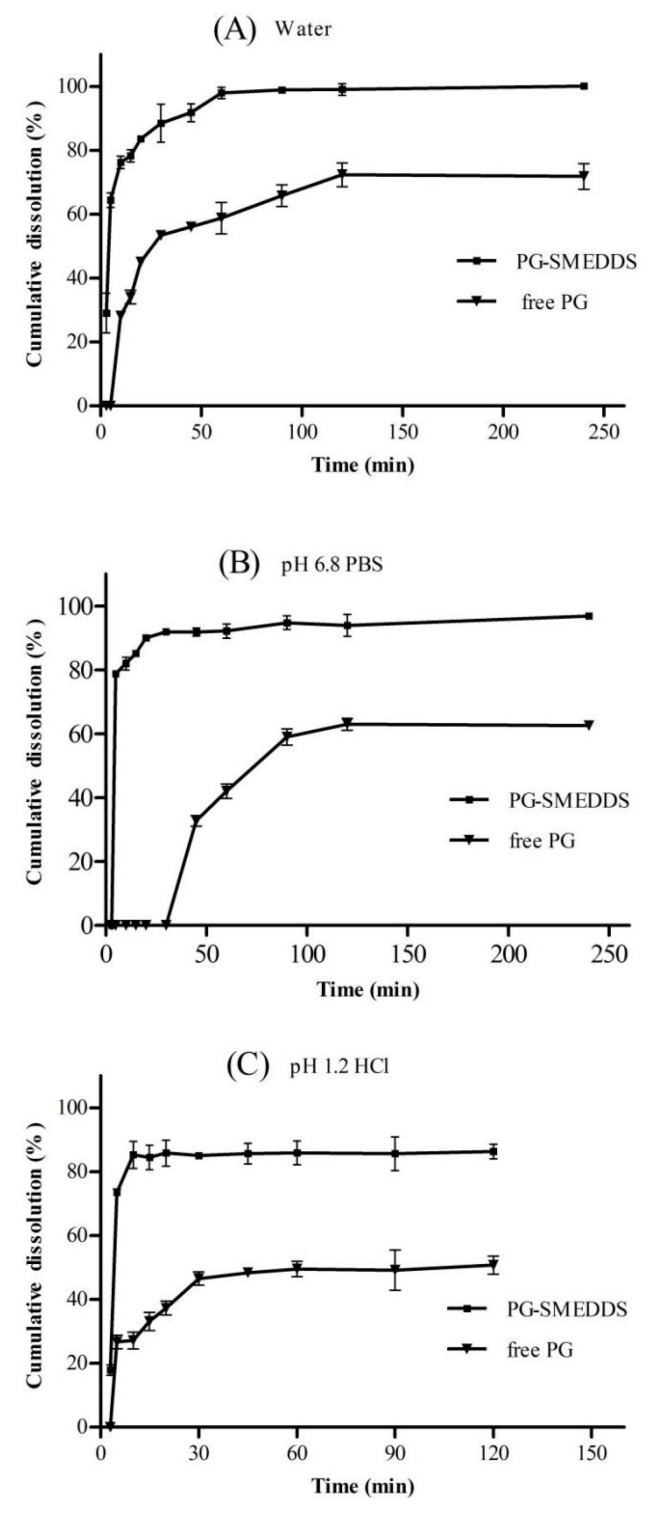
The dissolution profiles of PG-SMEDDS and free PG in different dissolution media (n = 3). (**A**) distilled water, (**B**) pH 6.8 PBS, and (**C**) pH 1.2 HCl.

The dissolution profiles of PG-SMEDDS and free PG in different aqueous media are depicted in Figure 6, indicating that the dissolution rate of PG-SMEDDS in the three different media was higher than that of free PG. In different dissolution media, the cumulative dissolution rate of PG-SMEDDS all reached more than 80% within about 15 min, but free PG only reached 40%. After 60 min, about 90% of PG was released from PG-SMEDDS in different media, and its dissolution rate was much higher than that of free PG. The results showed that PG-SMEDDS could significantly promote the rapid release of PG and increase the dissolution rate compared with the free drug. The rapid release of PG might be related to the formation of small droplets and their rapid dispersion. Besides, it was also evident that the release of PG from the SMEDDS was independent of pH.

### 3.7. Bioavailability Study in Rats

**Figure 7:**
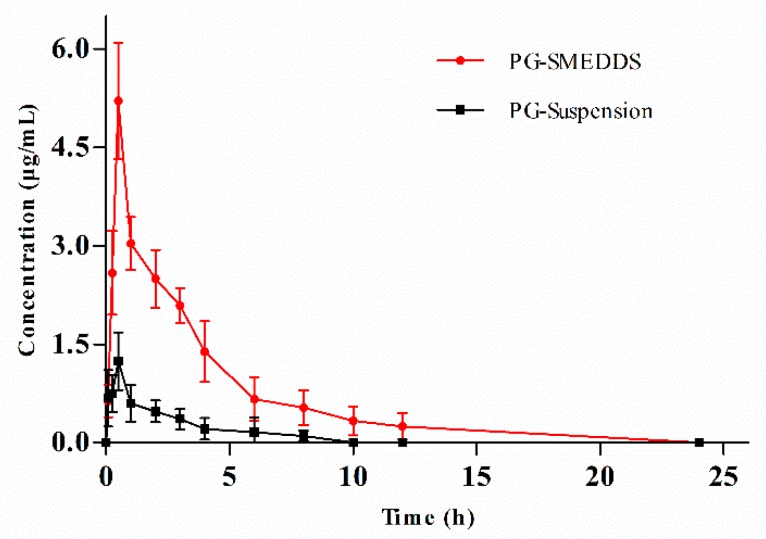
Plasma concentration-time profiles of PG in rats after oral administration of PG-SMEDDS and PG suspension (mean ± SD, n = 6).

After oral administration of PG-SMEDDS and PG suspension, the plasma concentration-time profiles of PG are shown in Figure 7. The profiles revealed that PG-SMEDDS could significantly improve the absorption of PG compared with the PG suspension. The main pharmacokinetic parameters of PG-SMEDDS and PG suspension calculated by non-compartmental model analysis are listed in Table 5. The time to reach maximum plasma concentration (T_max_) of PG-SMEDDS and PG suspension was 0.46 h and 0.5 h, while the maximum plasma concentration (C_max_) was 5.22 ± 0.87 mg/L and 1.24 ± 0.44 mg/L, respectively. T_max_ and C_max_ are important indicators that reflect the absorption degree of a drug in vivo. Although the T_max_ of PG-SMEDDS was not significantly improved, there was an approximately four-fold increase in C_max_. The results indicated that the absorption degree of PG-SMEDDS was better than that of the PG suspension in vivo. The increase of PG absorption degree might be related to the high dispersion and rapid emulsification of PG-SMEDDS under physiological conditions [32]. In addition, in comparison with the PG suspension, the mean residence time (MRT) and apparent half-life (T_1/2_) of PG-SMEDDS were increased significantly, which revealed that PG-SMEDDS could achieve the purpose of prolonging the action time of PG.

The relative bioavailability of PG-SMEDDS with respect to PG suspension was 587.77%, demonstrating that PG-SMEDDS was able to improve the oral absorption of the hydrophobic drug. The increase in bioavailability might be connected with the following factors: first, the surfactant in PG-SMEDDS could effectively increase the drug permeability through gastrointestinal tract membranes and promote transmembrane absorption [33]; second, the nano-sized droplets provided a large interfacial surface area for drug absorption and were also beneficial for increasing drug permeation across the intestinal membrane [34]. In summary, the prepared PG-SMEDDS could effectively improve the oral bioavailability of PG.

## 4. Conclusions

In order to increase the bioavailability of PG, the PG-SMEDDS formulation was developed. The optimal formulation of PG-SMEDDS was as following: 27.8% Labrafil M1944CS, 38.6% PEG-400, 33.6% Cremophor EL and 10.2 mg/g PG. The appearance of the developed PG-SMEDDS was clear, and the microemulsion droplets were spherical in shape with an average size of 40.11 ± 0.74 nm. The results of dissolution demonstrated that the cumulative dissolution rate of PG-SMEDDS could reach more than 90%. The main pharmacokinetic parameters suggested that the PG-SMEDDS could maintain long term plasma concentration. As a drug carrier, the developed SMEDDS successfully improved the oral absorption of PG and finally solved the problem of poor oral bioavailability of PG. Therefore, SMEDDS is a promising oral drug delivery system for improving the absorption and bioavailability of PG.

## Figures and Tables

**Table 1:** Factor level and the correspondent values of central composite design.

Independent Variable	Level				
	–1.414	–1	0	1	1.414
X_1_ (Oil%)	10	17.32	35	52.68	60
X_2_ (Km)	0.5	0.87	1.75	2.63	3

**Table 2:** Solubility of phillygenin (PG) in various oils, surfactants and co-surfactants (mean ± standard deviation (SD), n = 3). MCT: medium chain triglycerides; IPM: isopropyl myristate; TW80: Tween 80.

Types	Vehicles	Solubility (mg/g)
Oils	Olive oil	3.16 ± 0.10
	Ethyl oleate	2.54 ± 0.24
	Labrafil M1944CS	8.73 ± 0.18
	Medium chain triglycerides (MCT)	7.02 ± 0.02
	Isopropyl myristate (IPM)	2.02 ± 0.10
Surfactants	Cremophor RH40 (RH40)	48.63 ± 0.32
	Tween 80 (TW80)	58.55 ± 0.27
	Cremophor EL (EL-35)	70.34 ± 0.19
	P-octyl polyethylene glycol phenyl ether (OP-10)	60.46 ± 0.02
Co-surfactants	Transcutol HP	103.71 ± 0.1
	1,2-propanediol	9.67 ± 0.14
	Glycerol	0.12 ± 0.01
	Polyethylene glycol 400 (PEG-400)	92.75 ± 0.24

**Table 3:** Visual grading results of self-microemulsification efficiency.

Oils	Surfactants	Compatibility Ratio of Oil Phase and Surfactant						
		1:9	2:8	3:7	4:6	5:5	6:4	7:3
Labrafil M1944CS	EL-35	A	A	A	A	A	A	B
	OP-10	A	A	A	A	B	C	C
	Tween 80	A	A	B	C	C	C	C
MCT	EL-35	A	A	A	A	B	C	C
	OP-10	A	A	B	C	C	C	C
	Tween 80	A	A	B	C	C	C	C

**Table 4:** The results of central composite design. Km: weight ratio of surfactant to co-surfactant; PDI: polydispersity index.

Number	X_1_/Oil (%)	X_2_/Km	Y_1_/Equilibrium Solubility (mg/g)	Y_2_/Droplet Size (nm)	Y_3_/PDI
1	35	1.75	23.98	51.4	0.285
2	17.32	2.63	14.65	23.24	0.26
3	52.68	2.63	18.79	67.42	0.257
4	35	0.5	16.06	53.21	0.177
5	35	3	12.55	46.76	0.297
6	60	1.75	7.99	86.95	0.244
7	52.68	0.87	11.06	85.76	0.235
8	35	1.75	23.47	50.88	0.282
9	35	1.75	23.96	52.40	0.287
10	35	1.75	24.12	51.92	0.284
11	35	1.75	23.34	51.27	0.288
12	17.32	0.87	26.75	31.59	0.191
13	10	1.75	29.58	21.26	0.207

**Table 5:** Pharmacokinetic parameters of PG-SMEDDS and PG suspension in rats (mean ± SD, n = 6). C_max_: the maximum plasma concentration; T_max_: the time to reach maximum plasma concentration; AUC: area under the curve; MRT: mean residence time; T_1/2_: half-life; CL: Clearance.

Parameter	Unit	PG-SMEDDS	PG Suspension
C_max_	mg/L	5.22 ± 0.87 ***	1.24 ± 0.44
T_max_	h	0.46 ± 0.10	0.5 ± 0.00
AUC_(0-t)_	mg·h/L	16.34 ± 2.51 ***	2.78 ± 0.28
AUC_(0-∞)_	mg·h/L	16.43 ± 2.45 ***	2.78 ± 0.28
T_1/2_	h	2.49 ± 1.99	0.68 ± 0.18
MRT_(0-t)_	h	4.01 ± 0.75 **	2.69 ± 0.63
CL	L/h/kg	12.36 ± 1.52 ***	72.66 ± 7.23
Relative bioavailability	%	-	587.77

Compared to suspension, ** *P* < 0.01, *** *P* < 0.001.

## Supplementary Materials

The following are available online at https://www.mdpi.com/1999-4923/12/2/130/s1, Figure S1: HPLC chromatagrams of specificity (A: Reference substance solution; B: Sample solution; C: Blank solution), Figure S2: Diffusion results of different dyes (left: water-soluble dye; right: oil-soluble dye).

## References

[1] Qu H., Zhang Y., Chai X., Sun W. (2012). Isoforsythiaside, an antioxidant and antibacterial phenylethanoid glycoside isolated from Forsythia suspensa. Bioorg. Chem..

[2] Wang Z., Xia Q., Liu X., Liu W., Huang W., Mei X., Luo J., Shan M., Ma Z., Lin R. (2018). Phytochemistry, pharmacology, quality control and future research of Forsythia suspense (Thunb.) Vahl: A review. J. Ethnopharmacol.

[3] Shao S., Feng Z., Yang Y., Jiang J., zhang P. (2017). Eight new phenylethanoid glycoside derivatives possessing potential hepatoprotective activities from the fruits of Forsythia suspensa. Fitoterapia.

[4] Kicel A., Owczarek A., Michel P., Skalicka-Wozniak K., Kiss A.K., Olszewska M.A. (2015). Application of HPCCC, UHPLC-PDA-ESI-MS3 and HPLC-PDA methods for rapid, one-step preparative separation and quantification of rutin in *Forsythia* flowers. Ind. Corps Prod..

[5] Kuo P., Hung H., Nian C., Hwang T., Cheng J., Kuo D., Lee E.J., Tai S.H., Wu T. (2017). Chemical constituents and anti-inflammatory principles from the Fruits of *Forsythia suspensa*. J. Nat. Prod.

[6] Li C., Wei Q., Zou Z., Sun C., Wang Q., Zhao G., Yan X., Yu T., Gan C. (2019). A lignan and a lignan derivative from the fruit of *Forsythia suspensa*. Phytochem. Lett..

[7] Rahman M.M.A., Dewick P.M., Jackson D.E., Lucas J.A. (1990). Lignans of forsythia intermedia. Phytochemistry.

[8] Kang W., Wang J. (2009). In vitro antioxidant properties and in vivo lowering blood lipid of *Forsythia suspense* leaves. Med. Chem. Res..

[9] Su B., Zhu Q., Gao K., Yuan C., Jia Z. (1999). Lignan and Phenylpropanoid Glycosides from Lanceatibetica and Their Antitumor Activity. Planta Med..

[10] Liu W., Lu Y., Chu S., Jiang M., Bai G. (2019). Phillygenin, a lignan compound, inhibits hypertension by reducing PLCβ3-dependent Ca^2+^ oscillation. J. Funct. Foods.

[11] Song W., Wu J., Yu L., Peng Z. (2018). Evaluation of the Pharmacokinetics and Hepatoprotective Effects of Phillygenin in Mouse. BioMed Res. Int..

[12] Tang Y., Quan Y., Yu L., Zhen L., Li Y. (2019). Effect of Phillygenin on inflammatory response in LPS-induced RAW 264.7 cells. Nat. Prod. Res. Dev.

[13] Wu L., Qiao Y., Wang L., Guo J., Wang G., He W., Yin L., Zhao J. (2015). A Self-microemulsifying Drug Delivery System (SMEDDS) for a Novel Medicative Compound Against Depression: A Preparation and Bioavailability Study in Rats. AAPS PharmSciTech.

[14] Chen L., Liu C., Chen Q., Wang S., Xiong Y., Jing J., Lv J. (2017). Characterization, pharmacokinetics and tissue distribution of chlorogenic acid-loaded self-microemulsifying drug delivery system. Eur. J. Pharm. Sci..

[15] Yi T., Zhang J. (2019). Effects of Hydrophilic Carriers on Structural Transitions and In Vitro Properties of Solid Self-Microemulsifying Drug Delivery Systems. Pharmaceutics.

[16] Mahmoud D.B., Shukr M.H., Bendas E.R. (2014). In vitro and in vivo evaluation of self-nanoemulsifying drug delivery systems of cilostazol for oral and parenteral administration. Int. J. Pharm..

[17] Sermkaew N., Ketjinda W., Boonme P., Phadoongsombut N., Wiwattanapatapee R. (2013). Liquid and solid self-microemulsifying drug delivery systems for improving the oral bioavailability of andrographolide from a crude extract of *Andrographispaniculata*. Eur. J. Pharm. Sci..

[18] Dokania S., Joshi A.K. (2014). Self-microemulsifying drug delivery system (SMEDDS)-challenges and road ahead. Drug Deliv..

[19] Zhu Y., Xu W., Zhang J., Liao Y., Firempong C.K., Adu-Frimpong M., Deng W., Zang H., Yu J., Xu X. (2019). Self-microemulsifying Drug Delivery System for Improved Oral Delivery of Limonene: Preparation, Characterization, in vitro and in vivo Evaluation. AAPS PharmaSciTech.

[20] Qiao J., Ji D., Sun S., Zhang G., Liu X., Sun B., Guan Q. (2018). Oral Bioavailability and Lymphatic Transport of Pueraria Flavone-Loaded Self-Emulsifying Drug-Delivery Systems Containing Sodium Taurocholate in Rats. Pharmaceutics.

[21] Yao G., Li Y. (2011). Preparation, characterization and evaluation of self-microemulsifying drug delivery systems (SMEDDSs) of Ligusticum chuanxiong oil. Biomed. Preventive Nutr..

[22] Cui J., Yu B., Zhao Y., Zhu W., Li H., Lou H., Zhai G. (2009). Enhancement of oral absorption of curcumin by self-microemulsifying drug delivery systems. Int. J. Pharm.

[23] Vasconcelos T., Araújo F., Lopes C., Loureiro A., Neves J., Marques S., Sarmento B. (2019). Multicomponent self nano emulsifying delivery systems of resveratrol with enhanced pharmacokinetics profile. Eur. J. Pharm. Sci..

[24] Sharma P., Singh S.K., Pandey N.K., Rajesh S.Y., Bawa P., Kumar B., Gulati M., Singh S., Verma S., Yadav A.K. (2018). Impact of solid carriers and spray drying on pre/post-compression properties, dissolution rate and bioavailability of solid self-nanoemulsifying drug delivery system loaded with simvastatin. Powder Technol..

[25] Khoo S.M., Humberstone A.J., Porter C.J.H., Edwards G.A., Charman W.N. (1998). Formulation design and bioavailability assessment of lipidic self-emulsifying formulations of halofantrine. Int. J. Pharm..

[26] Yeom D.W., Song Y.S., Kim S.R., Lee S.G., Kang M.H., Lee S., Choi Y.W. (2015). Development and optimization of a self-microemulsifying drug delivery system for atorvastatin calcium by using D-optimal mixture design. Int. J. Nanomed.

[27] Patel A.R., Vavia P.R. (2007). Preparation and in vivo evaluation of SMEDDS (self-microemulsifying drug delivery system) containing fenofibrate. AAPS J..

[28] Zhang L., Zhang L., Zhang M., Pang Y., Li Z., Zhao A., Feng J. (2013). Self-emulsifying drug delivery system and the applications in herbal drugs. Drug Deliv..

[29] Liu W., Tian R., Hu W., Jia Y., Jiang H., Zhang J., Zhang L. (2012). Preparation and evaluation of self-microemulsifying drug delivery system of baicalein. Fitoterapia.

[30] Pharmacopoeia Commission of PRC (2015). Pharmacopoeia of the People’s Republic of China.

[31] Kang B.K., Lee J.S., Chon S.K., Jeong S.Y., Yuk S.H., Khang G., Lee H.B., Cho S.H. (2004). Development of self-microemulsifying drug delivery systems (SMEDDS) for oral bioavailability enhancement of simvastatin in beagle dogs. Int. J. Pharm.

[32] Djekic L., Janković J., Rašković A., Primorac M. (2018). Semisolid self-microemulsifying drug delivery systems (SMEDDSs): Effects on pharmacokinetics of acyclovir in rats. Eur. J. Pharm. Sci..

[33] Shah S.M., Jain A.S., Kaushik R., Nagarsenker M.S., Nerurkar M.J. (2014). Preclinical Formulations: Insight, Strategies, and Practical Considerations. AAPS PharmSciTech.

[34] Shah N.H., Carvajal M.T., Patel C.I., Infeld M.H., Malick A.W. (1994). Self-emulsifying drug delivery systems (SEDDS) with polyglycolyzed glycerides for improving in vitro dissolution and oral absorption of lipophilic drugs. Int. J. Pharm..

