# Supplementary material for: Self-Microemulsifying Drug Delivery System of Phillygenin: Formulation Development, Characterization and Pharmacokinetic Evaluation
Source: Pharmaceutics. 2020 Feb 3;12(2):130. doi: 10.3390/pharmaceutics12020130 (PMC7076376; doi:10.3390/pharmaceutics12020130)
Supplement: Supplementary file 1 [file pharmaceutics-12-00130-s001.pdf]

pharmaceutics

Supplementary Materials: Self-Microemulsifying Drug Delivery System of Phillygenin: Formulation Development, Characterization and Pharmacokinetic Evaluation

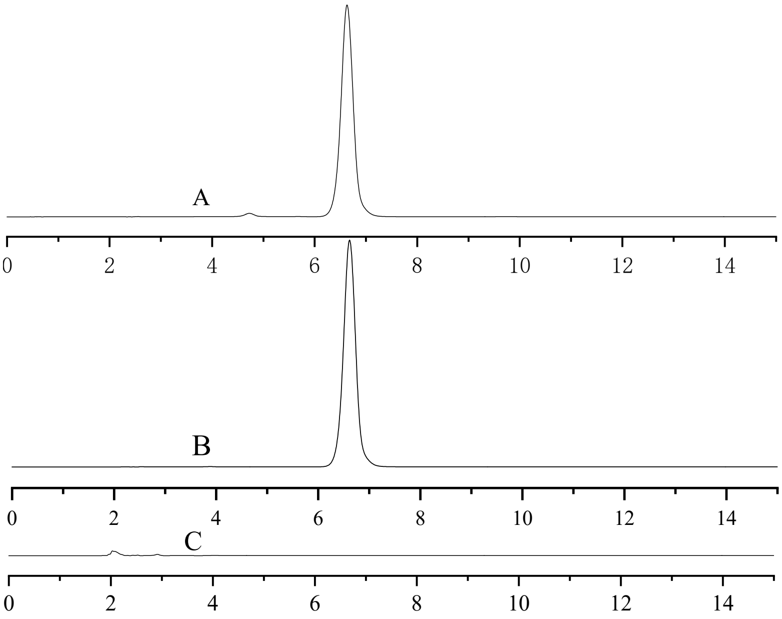

Figure S1. High Performance Liquid Chromatography (HPLC) chromatagrams of specificity (A: Reference substance solution; B: Sample solution; C: Blank solution).

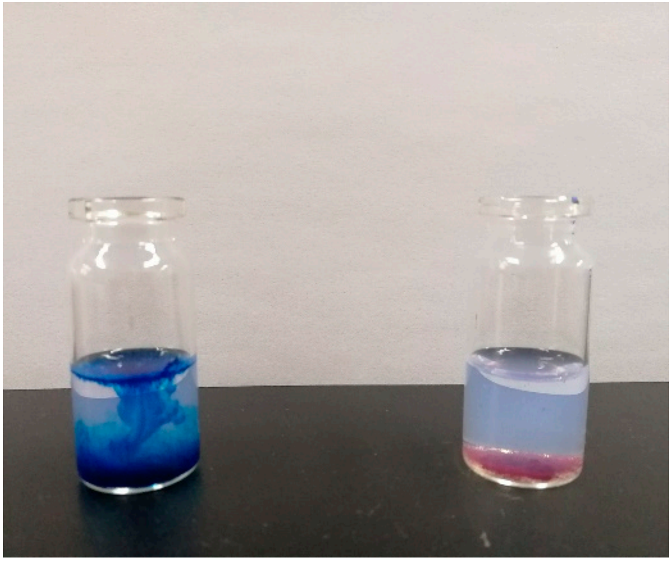

Figure S2. Diffusion result of different dyes (left: water-soluble dye; right: oil-soluble dye).
